# Case Report: Primary intravenous synovial sarcoma in the proximal great vein: a case report and literature review

**DOI:** 10.3389/fonc.2025.1634106

**Published:** 2025-09-05

**Authors:** Wenjian Tang, Zhen Wu, Rong Huang, Jiawang Wei

**Affiliations:** ^1^ Ganzhou Institute of Medical Imaging, Ganzhou Key Laboratory of Medical Imaging and Artificial Intelligence, Medical Imaging Center, Ganzhou People’s Hospital, The Affiliated Ganzhou Hospital of Nanchang University, Ganzhou, Jiangxi, China; ^2^ Department of pathology, Ganzhou People’s Hospital, The Affiliated Ganzhou Hospital of Nanchang University, Ganzhou, Jiangxi, China; ^3^ Department of Oncology, Ganzhou People’s Hospital, The Affiliated Ganzhou Hospital of Nanchang University, Ganzhou, Jiangxi, China

**Keywords:** primary, intravenous synovial sarcoma, proximal great vein, 18F-FDG PET/CT, case report

## Abstract

Primary intravenous synovial sarcoma (PISS) is an extremely rare disease originating from pluripotent mesenchymal stem cells, and has the potential to develop in nearly any part of the body. This report describes the case of a 54-year-old man who presented with a 20-day history of neck and facial swelling. Imaging findings on contrast-enhanced CT and ^18^F-FDG PET/CT revealed filling defects with markedly increased FDG uptake with involvement of multiple venous structures, including the superior vena cava, brachiocephalic vein, and left internal jugular vein, and enlarged lymph nodes near the aortic arch. A vascular interventional biopsy confirmed PISS. Here, we discuss the clinical characteristics and imaging findings of PISS and review 14 cases reported in previous studies.

## Introduction

Synovial sarcoma often occurs in deep soft tissues adjacent to large joints. However, synovial sarcoma does not arise from the synovium, it can occur almost anywhere in the body, and multipotent mesenchymal stem cells are considered the origin of malignant cells (1, 2). Synovial sarcoma arising in large veins represents a rare clinical entity. Here, we report a case of primary intravenous synovial sarcoma (PISS) in the proximal great vein.

## Case description

**Figure 1:**
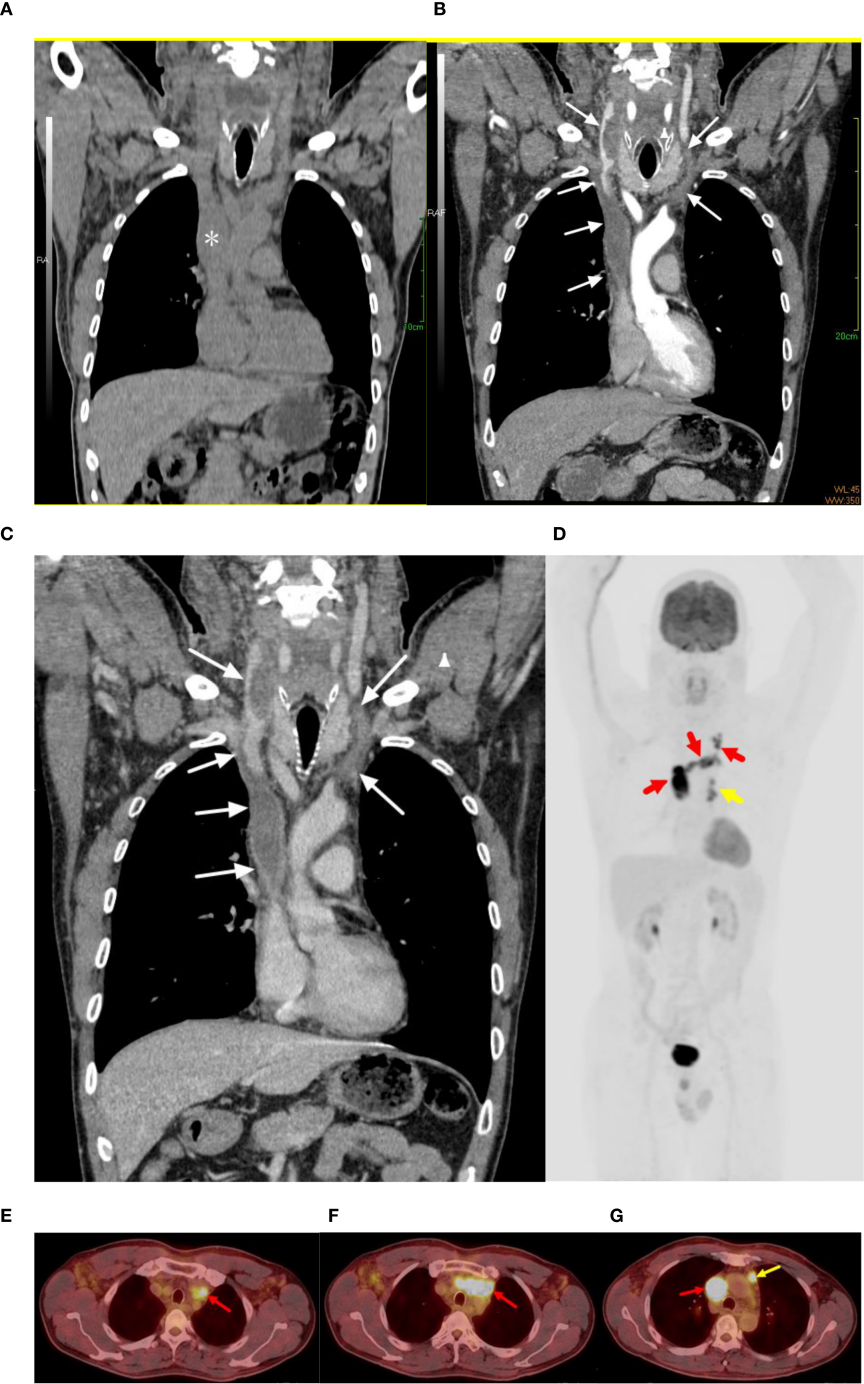
Contrast-enhanced CT and ^18^F-FDG PET images for the PISS patient. **(A–C)** Non-contrast CT reveals fusiform dilation of the superior vena cava. Contrast-enhanced CT revealed filling defects in the superior vena cava, brachiocephalic vein, and bilateral internal jugular vein (white arrows). **(D–G)** ^18^F-FDG PET/CT revealed intense FDG uptake in the superior vena cava, brachiocephalic vein, and left internal jugular vein (SUVmax 35.5; red arrows) and enlarged lymph nodes near the aortic arch with intense FDG uptake (SUVmax 13.7; yellow arrows). No significant FDG uptake is observed in the filling defect of the right internal jugular vein. These imaging findings definitively demonstrate that the filling defect observed in the right internal jugular vein results from thrombosis, whereas the filling defects identified in other regions are attributable to tumor tissue infiltration.

**Figure 2:**
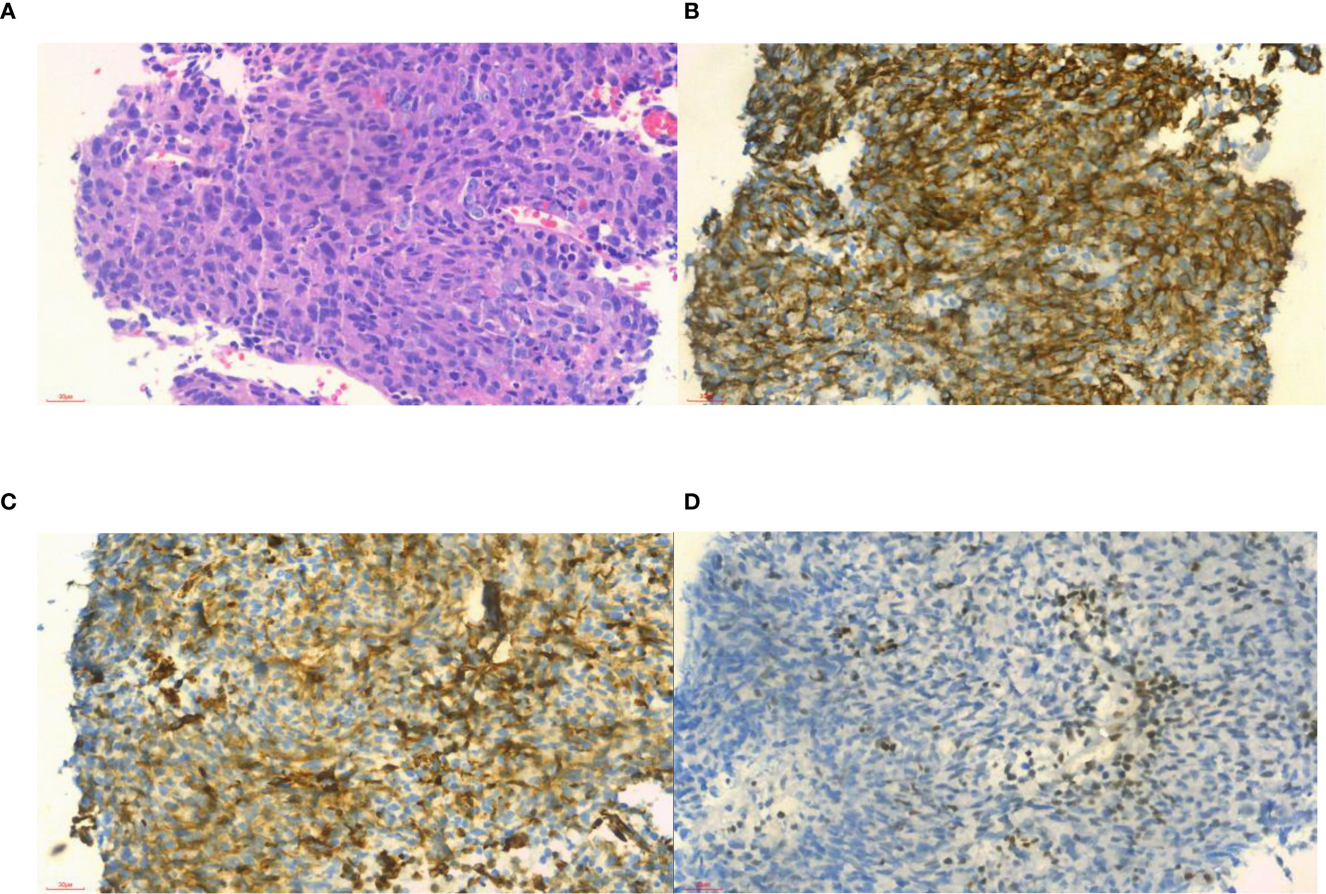
Histopathological and immunohistochemical examinations of samples from the patient. **(A)** Histologically, the tumor cells were composed of different proportions of epithelioid cells and spindle cells, suggesting a biphasic synovial sarcoma subtype (hematoxylin–eosin stain ×40); **(B)** positive in CK (×40); **(C)** positive in vimentin (× 40); and **(D)** focal positive in TLE1 (× 40).

A 54-year-old man presented with a 20-day history of neck and facial swelling. Physical examination revealed jugular vein and chest wall venous distention, indicating superior vena cava (SVC) syndrome. No positive signs were found in the joints of the limbs. Laboratory tests revealed a D-dimer concentration of 1.31 mg/L (reference range 0–0.55 mg/L). No abnormalities in the levels of tumor markers were detected. Non-contrast CT reveals fusiform dilation of the superior vena cava (**Figure 1A**, *). Contrast-enhanced CT (CECT) (**Figures 1B, C**, white arrow) revealed heterogeneously enhanced soft tissue masses with filling defects in the SVC, brachiocephalic vein, and left internal jugular vein. ^18^F-flurodeoxyglucose positron emission tomography/computed tomography (^18^F-FDG PET/CT) revealed intense FDG uptake (SUVmax 35.5; **Figures 1D–G**, red arrow) and enlarged lymph nodes near the aortic arch with intense FDG uptake (SUVmax 13.7; **Figure 1**, yellow arrow). No FDG uptake is observed in the filling defect of the right internal jugular vein. There was no evidence of distant metastasis. To alleviate symptoms, venous stenting was attempted via right femoral venipuncture; however, the surgery failed. Subsequently, an interventional biopsy of the lesion was performed. Histopathological examination of the lesion revealed synovial sarcoma (**Figure 2A**). Furthermore, the immunohistochemistry results revealed that the tumor cells were positive for CK, vimentin, TLE1 (**Figures 2B–D**), Fli-1 and β-catenin and negative for desmin, EMA, P63, S-100, SMA, CD31, and ERG, and the Ki-67 index was 80%. The patient was clinically diagnosed with primary intravenous synovial sarcoma (PISS) with mediastinal lymph node metastasis, leading to SVC obstruction syndrome. The patient subsequently underwent radiotherapy targeting the neck and upper mediastinum, which resulted in a reduction in neck and facial swelling. Imaging surveillance revealed no evidence of tumor progression during the 12-month follow-up. However, the patient subsequently committed suicide due to cancer-related depression.

## Discussion

PISS is an extremely rare disease originating from pluripotent mesenchymal stem cells. PISS exhibits a characteristic chromosomal translocation t(X; 18) (p11; q11), resulting in the formation of the SS18-SSX fusion genes (1, 2). We collected all the case reports related to PISS from the PubMed database. To our knowledge, only 14 cases have been reported in the literature (2–15), with a mean age at diagnosis of 38 years and a female-to-male ratio of 5:2 (**Table 1** in detail). Among these patients, eleven had lesions located in the inferior vena cava, iliac veins, femoral veins and lower extremity veins, whereas three had lesions located in the SVC, internal jugular vein and proximal subclavian vein. The clinical manifestations vary depending on the location of the disease. Lesions distal to the inferior vena cava typically manifest as lower extremity pain and swelling, whereas those involving the SVC primarily present with SVC obstruction syndrome. The prognosis of PISS is poor, with 1-year and 5-year survival rates of 45.3% and 11.5%, respectively (16). Furthermore, almost half of patients with synovial sarcoma experience local recurrence or metastasis after surgery (17).

**Table 1:** Clinical and imaging findings of primary intravenous synovial sarcoma reported in previous literature.

Ref.	Age/Sex	Symptom	Location	Imaging findings	Treatment	Outcomes
(2)	67/M	Superior vena cava (SVC) syndrome.	SVC and extending into the right atrium	The computed tomography (CT) and magnetic resonance imaging (MRI) findings were filling defects and heterogeneous delayed enhancement.	Palliative radiation and chemotherapy	Favorable results on subsequent CT and resolution of SVC syndrome.
(3)	34/F	pain in the left hip and swelling in the left leg.	Left wall of femoral vein	An extraskeletal calcification in the proximal thigh, and phlebography showed a thrombus in the femoral vein.	Surgery	A local recurrence was seen 5 years later, but the patient is still alive after 11 years follow-up.
(4)	31/F	Acute right abdominal pain and cardiac arrest.	Inferior vena cava (ICV), right atrium, right ventricle	Ultrasound (US) showed an mass in ICV. CT demonstrated obstruction arid dilatation of the ICV from the iliac bifurcation to the right atrium and embolism in bilateral pulmonary arteries.	Surgery	Postoperatively, the patient remained hypotensive and unconscious, and dead 1 day later.
(5)	34/F	Swelling in the left thigh with constant dull aching sensation.	Left femoral vein, and involved superficial femoral vein	MRI showed the mass to be predominantly solid with peripheral cystic areas.	Surgery, chemotherapy and radiotherapy	No evidence of recurrence.
(6)	56/F	swelling and pain in her right leg.	Right external iliac vein, with bilateral pulmonary emboli	US scanning confirmed thrombosis of the right common and external iliac veins. CT showed a soft tissue mass deforming the external iliac vein just proximal to the inguinal ligament.	Anticoagulant therapy, radiotherapy and surgery	Two years after surgery, she has had no local recurrence or systemic metastasis.
(7)	32/F	Pain and swelling in right arm.	SVC, right internal jugular vein, right proximal subclavian vein, protrusion into right atrium	MRI demonstrated that the tumor involved the entire SVC with extension into the right jugular vein, the distal subclavian vein, and the right atrium.	Surgery	lost to follow-up.
(8)	41/M	Sudden dyspnoea and right thigh swelling with sudden dyspnea.	Right superficial femoral vein, with popliteal vein thrombosis and bilateral pulmonary emboli.	US showed a highly vascularized lesion adherent to the femoral vascular bundle. Contrasted-enhanced CT (CECT) scan shows a solid mass at the level of the right lower thigh, contiguous with the superficial femoral artery.	Surgery and chemotherapy	After 1 year the patient remained well with no sign of recurrence or metastasis on CT scan.
(9)	32/F	Sudden dyspnea and swelling in right leg. Deep venous thrombosis and pulmonary embolism.	Right femoral vein	US showed thrombosis of the femoral and the popliteal veins and a heterogenous mass arising from the femoral vein invading the bifurcation of the femoral artery. CT confirmed the tumor in the right femoral vein.	Surgery, chemotherapy, anticoagulant therapy and compression stocking	Pulmonary metastasis occurred one year after the operation. One pulmonary lesion was resected, and no progression was observed 6 months later.
(10)	16/M	A 15-day history of dyspnea and palpitations.	SVC, right atrium	A chest radiograph showed cardiomegaly. A transthoracic echocardiogram confirmed a large mass filling the right atrium. The mass extended from the SVC to the tricuspid valve but did not prevent coaptation of the valve.	Surgery	The patient had an uneventful postoperative course.
(11)	41/F	progressive abdominal pain and distention, swelling in both legs, fatigue.	Inferior vena cava, right hepatic vein, right atrium	Abdominal US, CT and MRI revealed the presence of hepatomegaly, ascites, and mass arose from the retrohepatic portion of the inferior vena cava with extension above the diaphragm to the right atrium. A long occlusive thrombus extended from just below the tumor to the level of the renal veins.	Surgery, chemotherapy and anticoagulant therapy	Absent.
(12)	20/F	Progressive dyspnea for 3 days, deep-vein thrombosis 6 weeks earlier, pain in right leg for 12 months.	Right superficial femoral vein to common iliac vein, profound femoral vein, great saphenous vein	US and MRI found a mass in the right groin which compressed the femoral vein, and neoplastic structure was shown extending from the superficial femoral vein into the common iliac vein, profound femoral vein, and great saphenous vein. Additionally, thrombotic material was found attached to the neoplasm in the common iliac vein.	Anticoagulant treatment, surgery and chemotherapy	She suffered from sepsis after the operation and died of cardiogenic shock six months later. At the time of death, no tumor recurrence was evident.
(13)	49/F	Swelling and erythema in her left limb.	left superficial femoral vein	US showing a large intraluminal thrombus in the left superficial femoral vein, just adjacent to the femoral artery. CECT shows a heterogeneous nodular enhancement lesion in the lumen of the left superficial femoral vein, causing a venous thrombosis to extend up the common femoral and external iliac veins. MRI shows heterogeneous and progressive tumor enhancement, diffusion sequence shows restriction that suggests high cellularity.	Surgery, radiotherapy, and chemotherapy	The patient was disease-free for two years with strict medical visits and a very good quality of life.
(14)	26/M	Chest pain, a palpable and painful right inguinal mass, and edema in the right lower extremity.	Right external iliac vein and the right common femoral vein	US found a heterogeneous multilocular mass with well-circumscribed walls and hyperechoic internal septa, abundant blood flow signals in the mass. Plain CT scan of the lower abdomen showed multilocular soft-tissue mass with uneven density. CECT enabled confirmation of the presence of enhanced neo-vessels in the mass and heterogeneous enhancement of the mass with enhanced internal septa and unenhanced cystic areas.	Anticoagulants and surgery	Absent.
(15)	53/F	Right lower limb swelling and dilated veins over medial and anterior aspect of the thigh.	Right common femoral and external iliac veins, extending to the great saphenous vein	MRI and CT showed enhancing ovoid mass arising from the wall of the common femoral vein with small frond-like intraluminal projections with restricted diffusion. The lesion breaching the anterior wall of the vein. Non-enhancing thrombus was noted in the distal femoral and external iliac veins.	Surgery	Absent.

To the best of our knowledge, this study represents the first report of ¹^8^F-FDG PET/CT imaging in PISS, which provides significant clinical value for early disease diagnosis. PISS is often complicated by venous thrombosis and pulmonary embolism (14). Although CECT cannot definitively differentiate thrombotic from neoplastic filling defects, ¹^8^F-FDG PET provides discriminatory power based on their distinct metabolic profiles: thrombi demonstrate absent FDG uptake, contrasting with avid uptake in tumor tissue. Furthermore, the presence of calcifications, venous dilation with intraluminal cysts, fluid–fluid levels, and septations within a thrombosed vein on CECT warrant the consideration of intravascular tumors mimicking thrombosis (15). The enhancement of intravascular masses on CECT serves as a reliable diagnostic marker for tumors (15), providing a key feature for differentiating PISS from venous thrombosis. Therefore, CECT and ^18^F-FDG PET/CT contribute to the early diagnosis of PISS by enabling identification of tumor-thrombus boundaries, detection of potential lymph node metastases, and exclusion of secondary cancerous thrombi originating from other locations.

PISS needs to be differentiated from benign intravascular tumors and tumor-like lesions, such as intravenous pyogenic granuloma, intravascular fasciitis, masson’s tumor, leiomyoma, and lipoma. Intravascular lipoma presents as a distinct low-density mass on CT (18). Intravenous pyogenic granuloma, masson’s tumor and leiomyoma show only slight FDG uptake on ^18^F-FDG PET/CT, which is markedly lower than that of PISS (19–21). Intravascular fasciitis may present high FDG uptake, mimicking sarcoma (22). However, intravascular fasciitis predominantly involves the microvasculature, and is characterized by focal and small lesions. The differential diagnosis between PISS and other intravenous malignancies, such as intravenous leiomyosarcomas, intimal sarcomas and fat-poor liposarcomas, depends on pathology.

There is currently no standardized treatment for PISS. The management of PISS typically involves a multimodal therapeutic strategy including surgical resection, systemic chemotherapy, and radiation therapy. In cases of PISS complicated by thrombotic events, thrombolytic therapy is often indicated as a therapeutic strategy. Since the prevention strategies for synovial sarcoma are not well-defined, early detection and treatment are crucial.

## Conclusion

PISS is an exceptionally rare clinical entity. ​​This study presents the first documented characterization of ^18^F-FDG PET/CT imaging in PISS, while emphasizing the indispensable utility of CECT and ^18^F-FDG PET/CT for both diagnosis and differential diagnosis. Notably, PISS frequently presents with concomitant thrombosis, and the detection of malignant tumor components within a thrombotic background can significantly facilitate early diagnosis of PISS.

## Data Availability

The original contributions presented in the study are included in the article/supplementary material. Further inquiries can be directed to the corresponding author/s.
